# Challenges facing the More Doctors program (Programa Mais Médicos) in vulnerable and peri-urban areas in Greater Brasilia, Brazil

**DOI:** 10.1186/s12960-021-00672-2

**Published:** 2021-11-01

**Authors:** Helena Eri Shimizu, Leonor Maria Pacheco Santos, Mauro Niskier Sanchez, Thomas Hone, Christopher Millett, Matthew Harris

**Affiliations:** 1 Universidade de Brasília, Brasília, DF Brazil; 2 Imperial College, London, UK

**Keywords:** Human resources, Remote areas, Physicians, Primary health care, Vulnerable populations

## Abstract

**Background:**

A shortage of physicians, especially in vulnerable and peri-urban areas, is a global phenomenon that has serious implications for health systems, demanding policies to assure the provision and retention of health workers. The aim of this study was to analyze the strategies employed by the More Doctors Program (Programa Mais Médicos) to provide primary care physicians in vulnerable and peri-urban parts of Greater Brasilia.

**Methods:**

The study used a qualitative approach based on the precepts of social constructivism. Forty-nine semi-structured interviews were conducted: 24 with physicians employed as part of the More Doctors program, five with program medical supervisors, seven with secondary care physicians, twelve with primary care coordinators, and one federal administrator. The interviews occurred between March and September 2019. The transcripts of the interviews were submitted to thematic content analysis.

**Results:**

The partnership between the Ministry of Health and local authorities was essential for the provision of doctors—especially foreign doctors, most from Cuba, to assist vulnerable population groups previously without access to the health system. There was a notable presence of doctors with experience working with socioeconomically disadvantaged populations, which was important for gaining a better understanding of the effects of the endemic urban violence in the region. The incentives and other institutional support, such as enhanced salaries, training, and housing, transportation, and food allowances, were factors that helped provide a satisfactory working environment. However, the poor state of the infrastructure at some of the primary care units and limitations of the health service as a whole were factors that hampered the provision of comprehensive care, constituting a cause of dissatisfaction.

**Conclusions:**

More Doctors introduced a range of novel strategies that helped ensure a supply of primary care doctors in vulnerable and peri-urban parts of Greater Brasilia. The inclusion of foreign doctors, most from Cuba, was crucial for the success of the health services provided for the local communities, who subsist in violent and socioeconomically deprived urban areas. However, it became clear that barriers from within the health service itself hampered the physicians’ capacity to provide a satisfactory service. As such, what is needed for primary care to be effective is not just the recruitment, training, and deployment of doctors, but also investment in the organization of the whole health system.

**Supplementary Information:**

The online version contains supplementary material available at 10.1186/s12960-021-00672-2.

## Background

Peri-urban areas have difficulty retaining medical doctors, especially because of the precarious working conditions and network of services [1–5]. This is a challenge that must be overcome, through the creation of robust public policies designed to assure the access of the entire population to quality health services, leading to better health outcomes, as envisaged in the Sustainable Development Goals [1–6].

In Brazil, the public health system, Sistema Único de Saúde, has implemented a great many improvements [7], but still has trouble assuring equitable, comprehensive care to all [7] because of the scarcity and unequal distribution of primary health care (PHC) physicians [7], especially in far-flung and peri-urban areas, which are more socioeconomically deprived [7].

The More Doctors program (Programa Mais Médicos) was created in 2013, in response to a diagnostic analysis of the situation, which found that there were 1.8 physicians per 1000 inhabitants in Brazil—a number below that found in other countries with universal health coverage, such as Canada (2.4), the UK (2.7), Spain (3.5), and Portugal (3.8), and the mean of the 33 countries in the Organization for Economic Co-operation and Development [8, 9]. The program had a three-pronged approach: the emergency provision of doctors to work in areas, where such professionals were in short supply [10, 11], improvement of the infrastructure at PHC facilities, and the offer of more places in medical schools, under a new curriculum [10, 11].

By mid-2015, there were 18,240 professionals working as part of the More Doctors program in 81% municipalities in Brazil plus the federal district [11]. Most were Cuban (79.0%), followed by Brazilians licensed with the Regional Medicine Council (Conselho Regional de Medicina, CRM) (12.8%), Brazilians with degrees from abroad (3.1%), and doctors of other nationalities (5.1%) [11]. They were mostly deployed to more remote and vulnerable areas of the country, where the shortage of primary health physicians was most pronounced [10, 11]. The program also provided doctors for peri-urban areas, which had for many years coped with major deficiencies and/or an unreliable supply of physicians in their primary healthcare teams. These are transition areas between cities and the countryside, which often have unplanned urban developments with extremely vulnerable populations. They constitute a new type of multifunctional territory that has little in the way of institutional integration [12].

The creation of policies to assure the provision and retention of doctors in such areas is a challenge in many countries, especially given that these are areas, where health infrastructure is also lacking [13].

## The Greater Brasilia area and the More Doctors Program

**Fig. 1:**
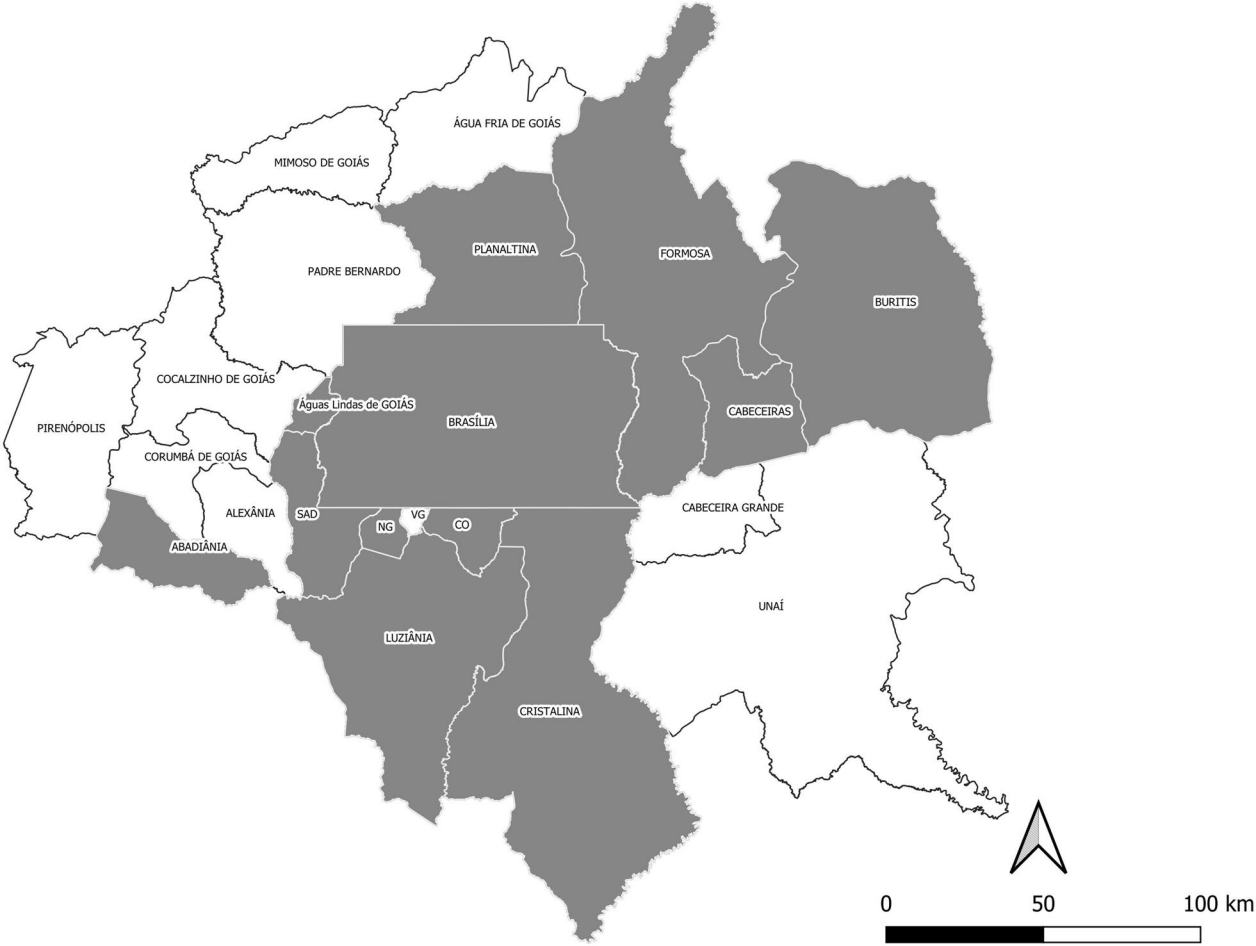
Study area—12 municipalities from The Integrated Development Region of the Federal District and Surrounding Area (RIDE). *SAD* Santo Antonio do Descoberto, *NG* Novo Gama, *VG* Valparaíso de Goiás, *CO* Cidade Ocidental

The study was conducted in Greater Brasilia (RIDE-DF region). This region is situated in the Central West region of Brazil and includes the federal district, nineteen municipalities from the state of Goiás, and two from Minas Gerais (Fig. 1).

These municipalities form the commuter belt around the capital city, Brasilia, made up of huge swathes of peri-urban and rural areas with a widely diverse socioeconomic and demographic profile. It, therefore, attracts migrant workers in search of better living conditions, who end up living in this commuter belt because of the high living costs in Brasilia itself.

In these municipalities, the coverage of the family health strategy in the primary care service ranges from 30 to 100%, while the population of the municipalities varies from around 4.1 million inhabitants. It also has around 400 health facilities and over 600 hospital beds. There is a large proportion of these population that use secondary and tertiary care in Brasília. The 2010 municipal human development index across the municipalities was more homogeneous (with the exception of Brasilia).

In the selected study area, the More Doctors program had a good performance in the “emergency provision of doctors”: most of the municipalities in the studied area received doctors as part of the program. Similarly, the opening of “new places for degree courses in medicine” happened in the larger city, Brasília, as expected. Indeed, the number of places more than doubled, from 304/year in 2012 (1 year before the program) to 668/year in 2019, mainly due to new two courses created at private universities, jointly offering 271 new places [14]. As far as “improvement of PHC units” is concerned, compliance was low, and the PHC infrastructure was considered inadequate in many municipalities.

The aim of this study was to analyze the strategies used by the More Doctors program, and whether they helped or hindered the provision of primary care doctors in vulnerable and peri-urban areas in municipalities from Greater Brasilia, according to actors who were actually involved in the program and, as such, are important bearers of knowledge, attitudes, and perceptions and are certainly influenced by the organizational capacity of the institution [15, 16]. This knowledge could be harnessed to improve policies for the provision and retention of primary care doctors in the short, medium, and long term [15, 16].

## Method

### Study design

This qualitative study is based epistemologically on the precepts of social constructivism [17, 18], which are often used to understand how different actors with knowledge about different elements of reality interact, which is valuable for policy analysis [17, 18].

Convenience sampling was used, and the inclusion criterion of the municipalities was that they must have hosted the More Doctors program for over 6 months. Twelve of the municipalities in the health region were accordingly included (Table 1).

**Table 1:** Characteristics of municipalities studied, Greater Brasilia, DF, 2019

State	Municipality	Population^1^	FHP^2^ coverage (%)	IDHM^3^
MG	Buritis	24,841	100	0.672
GO	Santo Antônio do Descoberto	74,744	84	0.665
GO	Formosa	121,617	62	0.744
GO	Cidade Ocidental	71,376	94	0.717
GO	Valparaíso de Goiás	168,468	67	0.746
GO	Planaltina	89,918	100	0.669
GO	Águas Lindas de Goiás	212,44	30	0.686
GO	Cristalina	58,997	78	0.699
GO	Cabeceiras	7993	100	0.668
GO	Novo Gama	115,711	61	0.684
GO	Abadiânia	20,042	88	0.689
DF	Brasília	3,015,268	41	0.824

^1^Population estimate, July 2019 (Instituto Brasileiro de Geografia e Estatística, IBGE)

^2^Estimated population covered by the Family Health Program

^3^Municipal Human Development Index, 2010 (Atlas de Desenvolvimento Humano do Brasil)

### Data collection

Forty-nine semi-structured interviews were conducted: one with a federal manager and the others in the twelve municipalities selected from the RIDE-DF region: 24 primary care physicians employed under the More Doctors program, five supervisors, seven secondary care physicians, and twelve primary care coordinators.

The interviews were held between March and September 2019. The interviewers received training to assure they administered the interview protocols in a standardized manner. There was a different script for each kind of interviewee to take account of their role in the process. The federal manager was asked about the criteria for the selection, recruitment, and deployment of the doctors, while for the others the following topics were covered: (1) details of previous education and inclusion in the More Doctors program; (2) experience working on the program; (3) work process in the program; (3) infrastructure at the primary care facility; and (4) organization of the primary care network and referral/counter-referral system.

The interviewees were free to respond as they wished and the interviews lasted 45 min on average. To complement this, the researchers used a field diary to observe the interactions with the interviewees and the conditions at the primary care units.

### Data analysis

The interview transcripts were analyzed using thematic analysis [17, 18], which meant coding the texts into themes and classifying them according to points of agreement and disagreement per theme. The themes themselves were developed until saturation was reached.

### Ethics and informed consent

This project is part of the multicenter study “Impact of Mais Medicos Program in Brazil”, which received approval from the research ethics committee of the Federal University of Paraíba (protocol no. 3.289.154). All the participants signed an informed consent form and their anonymity was assured by numbering the participants per type of profession.

## Results

Table 2 presents the profile of the health professionals who participated in the study. The profiles were quite similar, with most of them having less than 5 years of professional experience. The secondary care physicians were mostly male and had less than 5 year experience. The medical supervisors and the PHC coordinators (nurses) were mostly female and had more than 5 year experience. The federal manager interviewed was a male doctor and had more than 5 year previous experience.

**Table 2:** Description of health professionals interviewed in 12 municipalities in the peri-urban areas in Greater Brasilia, Brazil, 2019

Interviewee category	Number	Gender (%)		Type of diploma (%)		Previous experience (%)	
		Male	Female	Medical	Nurse	< 5 years	> 5 years
Primary health care doctor	24	50.0	50.0	100.0	0.0	62.5	37.5
Primary health care coordinator	12	8.3	91.7	25.0	75.0	8.3	91.7
Secondary health care doctor	07	71.4	28.6	100.0	0.0	71.4	28.6
Program medical supervisor	05	40.0	60.0	100.0	0.0	0.0	100.0
Program federal manager	01	100.0		100.0			100.0
Total	49						

## Factors related to the attraction and retention of doctors

The partnership between the Ministry of Health and the local authorities to implement the More Doctors program included a number of push factors to attract doctors. The factors the interviewees recognized as being strategic for getting doctors to move to these regions included the attractive employment conditions—better than those previously offered by the local authority—including the assurance of a regular paycheck:

I think that the conditions that More Doctors offers, financially, the support, and other stuff, it made doctors believe more in the program (Physician 11).

As part of the contracts, the doctors received housing, transport, and food allowances from the local authorities, which enabled them to rent a house near, where they worked and meant they were in closer contact with the local community.

When I came to the town, we get an allowance from the local authority, so I didn’t have any trouble in that respect. I was made really welcome. I get the housing allowance, the food allowance, all correct and on time. I don’t have any problem with it at all (Physician 6).

The interviewees indicated a number of pull factors that hampered the attraction and retention of doctors in the region. These included the location of the services in peripheral areas, a shortage of financial resources for the payment of the doctors’ wages, and the poor state of the health service infrastructure.

Places that aren’t attractive to professionals, like the outskirts of state capitals and other places. The municipality isn’t attractive for other professionals to come because wages aren’t always paid on time, materials they need are lacking. It’s not an enticing place for professionals, for a number of reasons. (Physician 22).

The precarious living conditions of the local people was also a reason for some doctors to move away. One of the interviewees indicated the great socioeconomic deprivation of the population and the poor state of the sanitation infrastructure and other public amenities.

Unless I’m mistaken, 40% of the population here don’t have sewage, basic sanitation. That’s just for starters. People’s homes are sometimes pretty ramshackle. The people here are very simple. Lots of them don’t know how to read (Physician 16).

## Pros and cons of having foreign doctors

As the program was unable to attract enough Brazilian physicians, professionals from Cuba were invited to the country, which posed some challenges. One of these was a certain disdain toward these physicians by the medical profession in Brazil, orchestrated by the CFM, responsible for regulating the approval of academic qualifications earned abroad.

They [the Cuban doctors] had those problems with the CFM, the medical lobby that rejected the arrival of the Cubans (Physician 22).

The Cubans’ language was also a barrier, because most of the service users do not understand Spanish. However, this was quickly compensated by their interpersonal skills—possibly developed during their medical training—which could be seen in the non-hierarchical way they dealt with the service users and the community.

One thing I think is really important about the professionals who came here with specific training, especially the ones who came from Cuba: their attitude when interacting with users, the position of their desk, not having the formal barrier of a table between doctor and patient. (Primary Care Coordinator 12).

There was a notable presence of doctors with experience working with populations who were socioeconomically disadvantaged. In particular, the Cuban doctors’ good interpersonal relations, resulting in good relations with the service users, local community, and health care team was a factor that imposed changes to the way primary care was provided. Demonstrations of sympathy, dialogue, and active listening were important for the forging of positive bonds:

The Cubans were concerned about the people. They weren’t just there to earn money. They actually came to work because of their love for people. (Primary Care Coordinator 5).

It was also noted that the Cuban doctors demonstrated resilience even when deployed to work in areas beset by different kinds of urban violence arising from social inequalities and a dearth of state institutions and services.

That’s how it works, the violence in the communities is worse than anything you can imagine. You learn to deal with violence, learn to deal with trafficking, with criminal factions, and then you manage to deal with healthcare. But you have to have this strong core if you’re going to manage to work (Physician 22).

## Training and continuous learning

The program offered the doctors training from the moment they arrived, giving short courses on the health system and the disease profile, given that most were foreign or had trained abroad. University involvement was important for the way the new professionals were received, providing graduate diploma courses in family health and supervision for doctors, as the following statement shows:

One great plus was this liaison with the universities, closing the gap between management and education. This cooperation with the universities, providing all the training and education (Primary Care Coordinator 12).

The graduate diploma course in family health was offered as a distance learning course and was designed to encourage the doctors to reflect on their own practice. Besides introducing them to the reality through the pedagogical strategy of micro-interventions, it gave them the opportunity to enhance the competences they would need when working in primary care.

My monograph for the graduate diploma course in family health was on reception at primary healthcare facilities and I applied it at the place where I worked (Physician 13).

The strategy of supporting the doctors through supervision with a view to improved primary health care practices was important for solving issues encountered in clinical practice. The use of online technologies was regarded as an important ancillary resource for resolving the problems encountered.

You can discuss things with your supervisor. It’s stimulating when someone asks you a question and you’re not completely sure about that subject, so you go away and research it to find out more. So I see it as positive. It was really good (Physician 7).

## Poor infrastructure at the primary health units and in the health system

The absence of adequate infrastructure was indicated as a barrier. Although some of the units had been renovated and some of their equipment had been replaced, this still posed a problem when it came to addressing the local people’s health issues.

The way I would evaluate primary care work—and not just here, but wherever I’ve worked—let’s say, the working conditions are precarious in every sense of the word. The logistics the local authority has to provide for you to work properly are faulty (Physician 1).

The precarious state of the health infrastructure was a cause of considerable anxiety on the part of the doctors, who perceived that this restricted the primary care system’s capacity to meet patient needs, often resulting in unnecessary referrals to secondary care.

Furthermore, it was found that in the vast majority of the municipalities, there were gaps in the health system coverage. It was also revealed that there was no counter-referral system: referrals were made, but specialized services such as psychiatry, surgery, or high-cost exams were in short supply. Consequently, the users had to wait a long time to get their problem resolved in the public health system or else never.

I mean, referral and counter-referral is terrible. I’ve been here five and a half years and I’ve never received a single counter-referral. So I get patients with no discharge summary, no counter-referral, and that makes my work harder because I have to believe what the patient tells me. (Physician 13).

## Discussion

More Doctors was instrumental in substantially increasing the provision of doctors for populations living in peri-urban areas, some of whom lived in conditions of extreme poverty [9–11] and had rarely or never had access to healthcare services in the outskirts of Brasilia.

Some of the strategies devised for the program constituted barriers and others facilitated it. These were analyzed in depth in this qualitative study. One of the limitations of this study is precisely the method it used, which did not enable the effects of the program to be analyzed. It was conducted in a region that straddles three states with vulnerable populations living in peri-urban areas. As such, it may not be representative of every area in the country. Furthermore, the fieldwork was conducted at the end of the program and the transition to the new program, Doctors for Brazil (Médicos pelo Brasil).

The medical staff employed via the program, which included a high proportion of Cubans, initially sparked some tensions in the peri-urban areas attended around the capital city, Brasilia [19–21], mainly because the local population and medical professionals had no previous experience of working with foreign doctors. However, despite the language barrier, these doctors quickly forged ties with the population, especially thanks to their previous experience of dealing with service users exposed to vulnerable socioeconomic conditions [10, 21, 22].

The collaboration for the provision of doctors was very effective and had positive impacts on the health of population groups traditionally excluded from access to the health system, because they either have no doctors or the provision of doctors is not reliable [22, 23].

Federal–municipal cooperation meant a framework of different strategies could be employed, which helped to attract and retain doctors in vulnerable and peri-urban areas [23, 24]. Wages were paid by the federal government, which was a great relief for the municipalities covered by the program, without which they would not have been able to achieve such a high level of medical coverage, since local authorities are already responsible for primary health care costs [23, 24], which constitute a considerable financial burden. The type of employment contract used, which offered good wages but was characterized as a scholarship, had the negative effect of making the labor conditions less stable [22, 25], since the doctors occupied a hybrid work/training space [8, 22, 25]. Despite the contractual weaknesses, the provision of wages that were compatible with the complexities and difficulties of the region constituted a positive aspect of the program [21, 22].

The local authorities were responsible for providing the doctors with housing, food, and travel allowances, and also for making sure they had enough free time to take part in the educational and training activities. These strategies were fundamental for retaining the doctors in close contact with the local communities, and even made them feel protective towards the vulnerable population groups they served, who were not only poor, but also lived side-by-side with urban violence, which has grown vertiginously in most of the municipalities in the RIDE-DF region, especially the ones closest to the capital.

The doctors interviewed as part of this study expressed great satisfaction with their graduate diploma course in family health, especially the methodology, which focused on connecting medical knowledge with the reality on the ground and thereby helped them solve some of the problems they faced in their daily work [25, 26]. Other studies have confirmed the positive effects of the courses, whether given in classroom settings or via distance learning, thanks to the possibility they provided for interaction, ameliorating the sense of isolation and other factors [27].

Supervision was another pillar on which the doctors could lean, which had a positive impact on their work, especially by helping them reach out to other members of the team and the local administration. Other studies have shown the positive effects of supervision, whether provided face-to-face, via technologies [26, 27], or in other ways that enable interaction with specialists, which allow a greater understanding of the roles, improve retention of health workers, and improve quality and confidence and encourage self-reflection [26–28].

One particular finding of our study was the approach the Cuban doctors employed in their work with the service users and local community and also with the other members of the health care team, fostering a more harmonious environment. The training of doctors to work in primary care should include issues relating to human rights and social justice with a view to reverting health inequities [29]. This mode of training is different from the purely biomedical paradigm, associated with increased dependence on technologies and the proliferation of specializations, which is one factor behind the limited interest physicians show in a career in primary care [29].

Besides the poor physical infrastructure at the primary health units, the doctors also struggled with gaps in the health care system, difficulties in referring users to other levels of care, and the non-existence of a counter-referral mechanism in most of the regions due to a lack of clear patient flows or the absence of a counter-referral culture among medical practitioners [30].

## Conclusion

More Doctors introduced a number of novel strategies that contributed to the provision of physicians at the primary care units in vulnerable and peri-urban parts of Greater Brasilia. The inclusion of foreign, mainly Cuban, doctors was fundamental for the approval of the health services by the local people, who subsist in conditions of great socio-economic deprivation and urban violence. The most important factors for attracting doctors to the region were the advantageous working conditions, including higher wages and the guarantee of a paycheck at the end of the month. Other important factors for attracting and retaining them in the region included the opportunity for continuous learning through a distance learning graduate diploma course and the support of supervisors. Nonetheless, it became clear that the barriers within the health system itself hampered the doctors’ work, demonstrating that what is needed for primary care to be effective is not just the recruitment, training, and deployment of doctors, but also investment in the organization of the whole health system (Additional files 1, 2).

## Supplementary Information

**Additional file 1:** Interview Script for Doctors from the More Doctors Program.

**Additional file 2:** Portuguese Abstract.

## Data Availability

The data generated, analysed and supported the findings of the current study are held by the Department of Collective Health of University of Brasília, but restrictions apply to the availability of this information, which were used under the ethics approval for the current study, and so are not publicly available.
