# Supplementary material for: Challenges facing the More Doctors program (Programa Mais Médicos) in vulnerable and peri-urban areas in Greater Brasilia, Brazil
Source: Hum Resour Health. 2021 Nov 1;19:134. doi: 10.1186/s12960-021-00672-2 (PMC8559374; doi:10.1186/s12960-021-00672-2)
Supplement: Supplementary file 1 — Additional file 1: Interview Script for Doctors from the More Doctors Program. [file 12960_2021_672_MOESM1_ESM.docx]

**Interview Script for Doctors from the More Doctors Program**

1. Could you tell me about your education and how you became involved in the More Doctors Program?
2. How was your arrival at the municipality – from your welcome (by the managers and community) to your integration with people at work and in the community?
3. What factors helped and hampered your experience in your working environment?
4. What factors helped and hampered your involvement in the community? (housing, food, support from society).
5. Could you tell me about how access to service users at your unit is organized?
6. Could you describe a standard week to show how the service is organized (programmatic actions)? How was it before? Did anything change after the More Doctors Program?
7. Do you have any time set aside to work in groups (health promotion activities), to have team meetings or to do home visits? How often do you do these activities?
8. How do you refer service users to get exams done?
9. How do you refer service users to get appointments with specialists? What has your experience of referral and back-referral been like? Do you always refer your patients? Do you get any feedback on patients you refer (back-referral)?
10. Did you notice any change in the working process after you arrived at the municipality? If so, what differences did you notice at your unit and in the municipal health care network before and after your arrival as part of the program?
11. Were any changes to teamwork made after your arrival? Were any renovations done or was any equipment acquired for the primary health unit after you joined with the More Doctors program?
12. In what way did the supervision process contribute to the work at the primary health unit?

**Interview Script for Primary Health Care Coordinators**

1. How was the arrival of the doctor as part of the More Doctors Program (MDP) to your municipality?
2. Could you tell me a bit about your experience with the MDP?
3. How has your working process with the MDP doctor been?
4. Did you notice any differences between the MDP doctors and the doctors who were already working there in terms of integration, participation in different activities at the unit, and teamwork?
5. How many hours does the doctor spend at the unit at the moment? How did it use to be?
6. What factors helped and hampered the work of the foreign doctor?
7. How is access to the municipality’s and region’s health care system organized? What about the referral and back-referral systems?
8. Have there been any renovations done and/or acquisition of equipment or materials for the primary health care unit since your arrival with the MDP?

**Interview Script for Supervisors of Doctors Contracted as part of the More Doctors Program**

1. In what way did the supervision process contribute to the work at the primary health care unit?
2. How do patients make appointments? Did this change after the arrival of the More Doctors Program (MDP)?
3. Has there been any change, before and since the introduction of the MDP, in the time patients have to wait for a doctor’s appointment and the length of a consultation?
4. Do you and the team have allotted times or days when you can work together as a group, have team meetings, and do home visits? Has this changed since the arrival of the doctor with the MDP?
5. What different health services are there at your municipality?
6. How do you make appointments for exams and specialists?
7. Do you get any feedback on patients that have received referrals (back-referral)?
8. How do you think your unit and the municipal health system have changed since the arrival of the MDP?

**Interview Script for the Federal Administrator**

1. Could you tell me about the criteria for the selection, recruitment, and deployment of the doctors in Brazil?
2. Could you explain what challenges and difficulties there were in providing doctors for remote and socioeconomically vulnerable areas?
