# Supplementary material for: Challenges facing the More Doctors program (Programa Mais Médicos) in vulnerable and peri-urban areas in Greater Brasilia, Brazil
Source: Hum Resour Health. 2021 Nov 1;19:134. doi: 10.1186/s12960-021-00672-2 (PMC8559374; doi:10.1186/s12960-021-00672-2)
Supplement: Supplementary file 2 — Additional file 2: Portuguese Abstract. [file 12960_2021_672_MOESM2_ESM.docx]

**Portuguese Abstract**

Introdução: A escassez de médicos, sobretudo em áreas periurbanas e vulneráveis, caracteriza-se como um fenômeno global, com sérias implicações para os sistemas de saúde, exigindo políticas que garantam o seu provimento e fixação. Este estudo teve como objetivo analisar as estratégias utilizadas pelo Programa Mais Médicos (PMM), que se constituíram em fatores facilitadores na provisão de médicos na Atenção Primária, em áreas periurbanas e vulneráveis dos municípios da Região Integrada do Distrito Federal e Entorno-Brasil.

Métodos: Estudo qualitativo fundamentada no construtivismo social. Foram realizadas 49 entrevistas semiestruturadas com profissionais, sendo 24 médicos do PMM, cinco supervisores médicos do PMM, sete médicos da Atenção Secundária, 12 coordenadores da APS, e uma entrevista com gestor federal, entre março e setembro de 2019, que foram submetidas à análise temática de conteúdo.

Resultados: A parceria entre o Ministério da Saúde e os municípios foi fundamental para prover médicos para a região, principalmente com médicos estrangeiros, a maioria de Cuba, possibilitando o acesso aos serviços de saúde às populações vulneráveis, antes excluídas do sistema. O perfil dos médicos com experiência de trabalho com populações com vulnerabilidades socioeconômica mostrou-se profícuo, inclusive para a melhor compreensão e enfrentamento da violência urbana comumente presente na região. Os incentivos e outros apoios institucionais como salário mais alto, treinamento, ajuda para moradia, transporte e alimentação, apresentaram-se como fatores que contribuíram para um clima satisfatório no trabalho; todavia a precariedade da infraestrutura em algumas unidades, e os fatores relacionados às insuficiências do sistema, caracterizaram-se como fatores que dificultam a integralidade do cuidado, constituindo-se causa de insatisfação.

Conclusões: O PMM implementou diversas estratégias inovadoras que contribuíram para a provisão dos médicos nas unidades de APS das regiões periurbanas e vulneráveis da RIDE-DF. A composição com médicos estrangeiros: os cubanos foram fundamentais para qualificar as ações de saúde desenvolvidas junto as comunidades, que convivem com grandes privações sócio econômicas e violência urbana. Todavia, ficou evidente que as barreiras relacionadas ao sistema de saúde, dificultaram a atuação do médico, demonstrando que para produzir APS eficiente e eficaz, é necessário, além de recrutar, treinar e implantar médicos, investir na melhor organização da rede de atenção à saúde.

Palavras chave: Recursos humanos, Zonas remotas, Médicos, Atenção Primária à Saúde, Populações Vulneráveis.
